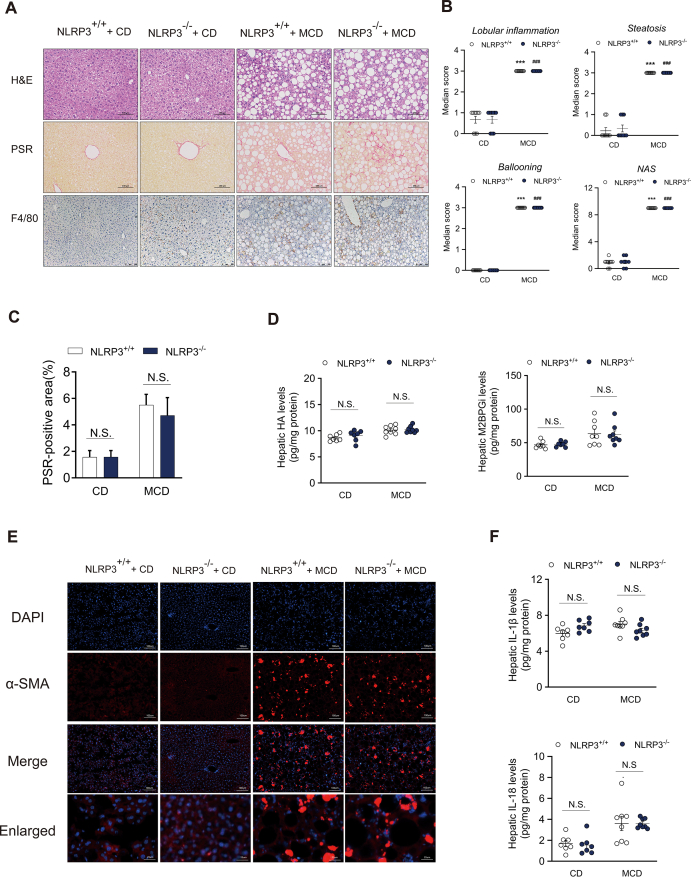# NLRP3 deficiency reprograms hepatic glucose metabolism but fails to ameliorate hepatic inflammation and fibrosis in the MCD model

**DOI:** 10.1016/j.gendis.2025.101921

**Published:** 2025-11-06

**Authors:** Yong Zou, Xiaowen Wu, Jingyi Wang, Jie Xia, Sen Zhang, Shuzhe Ding, Weina Liu, Zhengtang Qi

**Affiliations:** a Key Laboratory of Adolescent Health Assessment and Exercise Intervention of the Ministry of Education, East China Normal University, Shanghai 200241, China; b College of Physical Education and Health, East China Normal University, Shanghai 200241, China; c Department of Physical Education, Shanghai Jiao Tong University, Shanghai 200240, China

NLR family pyrin domain-containing 3 (Nlrp3) inflammasome, known for its role in mediating inflammatory responses at cellular and tissue levels, has now emerged as a pivotal mediator in the pathogenesis of metabolic disorders.^1^ In models of ageing and obesity-related metabolic disorders, Nlrp3 manipulation has been demonstrated to correlate with the maintenance of glucose metabolic homeostasis.^2^^,^^3^ Both systemic and liver-specific Nlrp3 knockout mouse models demonstrate a critical role of Nlrp3 in the regulation of the hepatic insulin signaling pathway.^4^ Given the liver's pivotal role in glucose homeostasis, it is plausible that Nlrp3 contributes to the modulation of hepatic glucose metabolism; however, a comprehensive mapping of this metabolic network remains incompletely characterized. In a different context, Nlrp3-specific inhibitors have been demonstrated to attenuate methionine and choline-deficient diet (MCD)-induced hepatic fibrosis and inflammation,^5^ whether genetic ablation of Nlrp3 confers protective effects remains to be elucidated. Therefore, this study was conducted to investigate the role of Nlrp3 in hepatic glucose metabolism, and additionally, to evaluate its potential protective effects in an MCD-induced mouse model.

Global NLRP3 knockout (Nlrp3^−/−^) mice were first generated using CRISPR technology, and deletion efficiency in the liver was confirmed by Western blotting (Fig. S1A). Nlrp3^−/−^ mice exhibited elevated fasting but reduced postprandial blood glucose levels (Fig. 1A and B), indicative of disrupted glucose regulation. To exclude potential developmental compensation and systemic metabolic confounders, we further assessed metabolic phenotypes in Nlrp3^−/−^ mice. Compared with their littermate controls, Nlrp3^−/−^ mice showed no significant difference in total body weight (Fig. S1B). The Nlrp3^−/−^ mice exhibited reduced lean mass and increased fat mass (Fig. S1C and S1D), accompanied by decreased muscle tissue mass and elevated white adipose tissue mass (Fig. S1E and S1F). These alterations in organ composition, particularly in muscle, a key glucose-consuming organ, did not lead to significant differences in diurnal energy expenditure, food intake, or physical activity levels (Fig. S1G–S1J). Furthermore, glucose tolerance and insulin sensitivity remained unaffected (Fig. S1K and S1L). This suggests that glucose regulation in other organs, like the liver, may play a potential role in the blood glucose fluctuations observed in Nlrp3^−/−^ mice across different feeding states.

**Figure 1:**
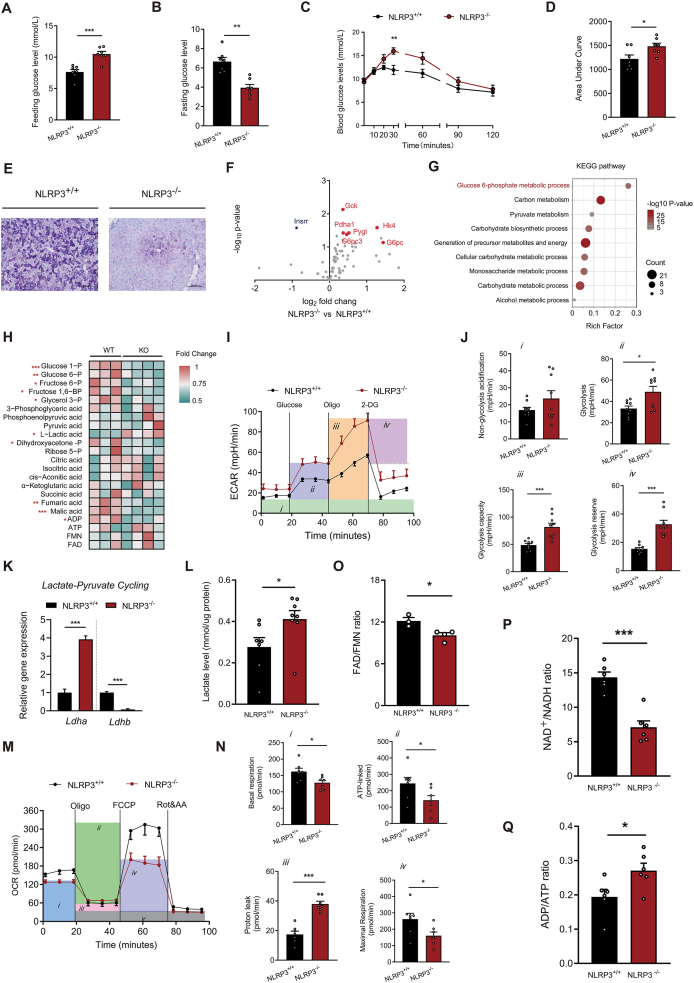
Nlrp3 deficiency fails to protect against choline-deficient diet-induced liver injury but alters hepatic glucose flux. **(A, B)** Blood glucose levels in fed (B) and fasted (B) states in Nlrp3^−/−^ male mice and controls (*n* = 7 per group). **(C, D)** Pyruvate tolerance test (C) and its AUC (D) performed in male mice and controls (*n* = 7 per group). **(E)** Representative PAS-stained liver sections showing glycogen accumulation (intense magenta signal) in Nlrp3^−/−^ and control mice (*n* = 3 per group). **(F, G)** The volcano plot showing differentially expressed genes related to glucose metabolism (F) and KEGG pathway enrichment analysis (G) comparing Nlrp3^−/−^ mice and controls (*n* = 5 per group). **(H)** Relative abundance of central carbon metabolites, normalized by the mean of the WT group (*n* = 3–4 per group). **(I)** Relative extracellular acidification rate (ECAR), indicating glycolytic activity in primary hepatocytes from Nlrp3^−/−^ and control mice (*n* = 9 per group). **(J)** Glycolytic activity and glycolysis capacity in primary hepatocytes from Nlrp3^−/−^ and control mice (*n* = 9 per group). **(K)** Relative mRNA expression of Ldha and Ldhb in primary hepatocytes, reflecting alterations in the pyruvate-lactate shuttle (*n* = 7 per group). **(L)** Intracellular lactate levels in primary hepatocytes from Nlrp3^−/−^ and control mice (*n* = 7 per group). **(M)** Relative oxygen consumption (OCR), indicating aerobic metabolism activity in primary hepatocytes from Nlrp3^−/−^ and control mice (*n* = 7 per group). **(N)** Aerobic metabolism activity and mitochondria metabolism capacity in primary hepatocytes from Nlrp3^−/−^ and control mice (*n* = 7 per group). **(O)** Hepatic FMN/FAD ratio in Nlrp3^−/−^ and control mice based on untargeted metabolomics data (*n* = 3–4 per group). **(P)** Intracellular NAD^+^/NADH ratio in primary hepatocytes from Nlrp3^−/−^ and control mice (*n* = 6 per group). **(Q)** Intracellular ADP/ATP ratio in primary hepatocytes from Nlrp3^−/−^ and control mice (*n* = 6 per group). The data were expressed as mean ± standard error. ∗*P* < 0.05, ∗∗*P* < 0.01, and ∗∗∗*P* < 0.001 versus the control.

To further explore this phenotype, we performed pyruvate tolerance tests, which revealed enhanced hepatic gluconeogenic capacity in Nlrp3^−/−^ mice (Fig. 1C and D). However, glycogen accumulation in the liver was markedly reduced (Fig. 1E). These seemingly paradoxical results suggest a potential metabolic rewiring of glucose metabolism in the livers of Nlrp3^−/−^ mice. To investigate the hepatic glucose metabolism network driven by NLRP3 deficiency, we performed a PCR array to compare relevant gene expression in the liver of Nlrp3^−/−^ and control mice. We observed up-regulation of key genes involved in hepatic glycogenolysis, including liver glycogen phosphorylase (*Pygl*) and glucose-6-phosphatase (*G6pc*), consistent with our earlier findings of elevated blood glucose levels and reduced hepatic glycogen stores. Concurrently, key glycolytic genes, such as hexokinase (*Hk3*), glucokinase (*Gck*), phosphofructokinases (*Pfkl*), and lactate dehydrogenase A (*Ldha*), were also up-regulated (Fig. 1F). Kyoto Encyclopedia of Genes and Genomes (KEGG) enrichment analysis of differentially expressed genes further revealed significant enrichment in metabolic pathways involving glucose-6-phosphate (G6P), which is a crucial hub intermediate metabolite between glycogenolytic and glycolytic pathways (Fig. 1G). This indicates that the livers of Nlrp3^−/−^ mice are more inclined to channel glucose toward catabolic metabolism rather than store it as glycogen. To further determine whether the changes we observed at the transcriptional level translate to alterations in intermediate metabolites of glucose metabolism in the liver, we employed targeted metabolomics focusing on central carbon metabolism to quantify key metabolites in these pathways. Nlrp3^−/−^ mice liver exhibited a marked consumption of glycolytic intermediates (G-6-P, F-6-BP, F-1,6-BP) alongside elevated lactate during glucose mobilization (Fig. 1H; Fig. S1M). Furthermore, the abundances of TCA cycle intermediates, such as fumarate and malate, were reduced, indicating a concomitant impairment in aerobic mitochondrial metabolism (Fig. 1H; Fig. S1M). Transcriptomic and metabolic profiles indicate that Nlrp3 deficiency up-regulates rate-limiting glycolytic enzymes, enhancing catabolic output and glucose anaerobic metabolism.

To validate our observations in hepatocytes, we isolated primary hepatocytes from mice for further analysis. Nlrp3 deficiency led to enhanced glycolytic activity in hepatocytes (Fig. 1I), including basal glycolysis, glycolytic capacity, and glycolysis reserve (Fig. 1J). To corroborate these functional data, we performed quantitative PCR analysis, which showed up-regulated expression of lactate dehydrogenase isoforms *Ldha* and *Ldhb*, key enzymes facilitating the interconversion between pyruvate and lactate. Consistently, lactate measurements in hepatocyte culture media demonstrated elevated lactate production in Nlrp3^−/−^ cells compared with controls (Fig. 1K and L). We also assessed the mitochondrial oxidation capacity in primary hepatocytes isolated from Nlrp3^−/−^ mice. Consistent with our *in vivo* metabolic profiling, NLRP3 deficiency significantly impaired mitochondrial oxidation (Fig. 1M), including basal respiration, maximum respiration capacity, and ATP-linked respiration, but with more proton leaking (Fig. 1N; Fig. S1N). Metabolic reprogramming in cells is typically associated with alterations in cellular energy status. We calculated the FMN/FAD ratio in mouse livers and observed a decrease in NLRP3 knockout mice (Fig. 1O), which is consistent with the reduced mitochondrial oxidative function in hepatocytes identified in our study. Concurrently, the NAD^+^/NADH ratio was decreased while the ADP/ATP ratio was increased in primary hepatocytes from Nlrp3^−/−^ mice (Fig. 1P). These findings suggest that NLRP3 deficiency leads to hepatic glucose metabolic reprogramming, likely due to cellular energy insufficiency and impaired mitochondrial oxidative capacity. Therefore, our experimental results demonstrate that Nlrp3 deficiency disrupts systemic glucose homeostasis, potentially through metabolic rewiring of hepatic glucose metabolism. Specifically, Nlrp3 deletion shifts the liver toward enhanced glucose catabolism rather than glycogen synthesis, and reduces aerobic oxidation while promoting anaerobic glycolysis.

Separately, to investigate the role of NLRP3 in hepatic inflammation and fibrosis, we subjected Nlrp3^−/−^ mice to the MCD model. After 6 weeks of MCD feeding, we employed ultrasonography to assess hepatic structure and hemodynamics *in vivo*. MCD feeding led to reduced portal vein diameter, narrowing of the inferior vena cava, and increased liver parenchymal echogenicity, features indicative of fibrotic remodeling (Fig. S2A and S2B). However, NLRP3 deficiency failed to reverse these morphological alterations, which was further supported by unchanged serum ALT and AST levels (Fig. S2A and S3C). We also observed that browning of the liver and increased liver weight persisted in Nlrp3^−/−^ mice following MCD feeding (Fig. S3A and S3B). To further validate our findings, we performed histological analysis of liver tissues following MCD feeding. This revealed increased hepatic steatosis, ballooning, inflammation, and elevated NAS scores, all of which remained unchanged in Nlrp3^−/−^ mice (Fig. S4A and S4B). Sirius red staining and fibrosis-related ELISA markers confirmed enhanced fibrosis under MCD diet, with no alleviation by NLRP3 deficiency (Fig. S4C and S4D). Similarly, MCD-induced inflammation up-regulation, such as macrophage infiltration and secreted IL-1β and IL-18, as well as α-SMA-positive stellate cell activation, remained unaltered in Nlrp3^−/−^ mice (Fig. S4E and S4F). Together, these findings demonstrate that NLRP3 deficiency does not protect against MCD-induced hepatic injury, inflammation, or fibrosis.

In summary, our results demonstrate that NLRP3 deficiency reprograms hepatic glucose metabolism by enhancing anaerobic glycolysis, which may be attributed to impaired aerobic metabolic capacity and consequent energy shortage in the liver. Moreover, NLRP3 deficiency did not confer protection against MCD diet-induced hepatic inflammation or fibrosis in mice. Together, these findings highlight a novel role for Nlrp3 in coordinating hepatic glucose metabolism and systemic glucose balance.

The MCD diet model exhibits pathological disparities from human metabolic-associated steatohepatitis, which limits its clinical translatability. Additionally, some experiments involved a relatively small sample size. In future studies, hepatocyte-specific NLRP3 knockout and rescue experiments will be performed to further elucidate the role of NLRP3 in regulating hepatic glucose metabolism.

## CRediT authorship contribution statement

**Yong Zou:** Writing – original draft, Validation, Software, Project administration, Methodology, Formal analysis, Data curation, Conceptualization. **Xiaowen Wu:** Validation, Investigation, Formal analysis, Data curation. **Jingyi Wang:** Validation, Software, Investigation, Formal analysis, Data curation. **Jie Xia:** Writing – review & editing, Supervision, Software, Resources, Conceptualization. **Sen Zhang:** Writing – review & editing, Investigation, Conceptualization. **Shuzhe Ding:** Supervision, Resources, Project administration, Investigation, Conceptualization. **Weina Liu:** Supervision, Resources, Project administration, Investigation, Funding acquisition. **Zhengtang Qi:** Writing – review & editing, Supervision, Resources, Project administration, Investigation.

## Ethics declaration

Animal protocols were reviewed and approved by the Animal Care and Use Committee of East China Normal University (approval number: 20210132).

## Funding

This study was supported by the 10.13039/501100001809National Natural Science Foundation of China (No. 32271174, 32571316) and the Key Program of National Social Science Fund of China (No. 23AZD092).

## Conflict of interests

The authors declared no conflict of interests.

## Figures and Tables

**figs1:**
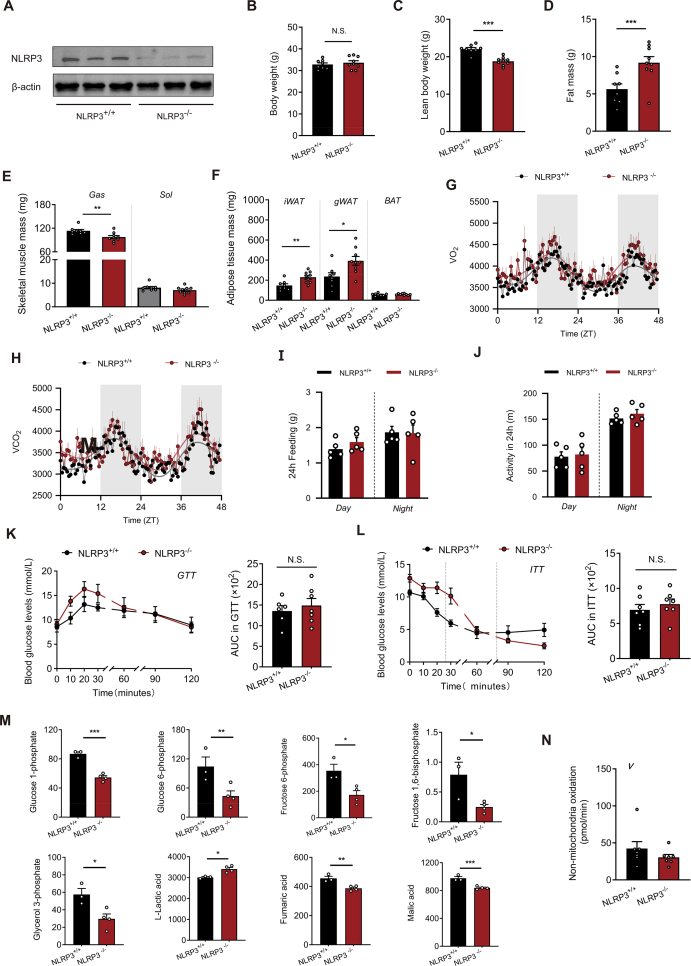


**figs2:**
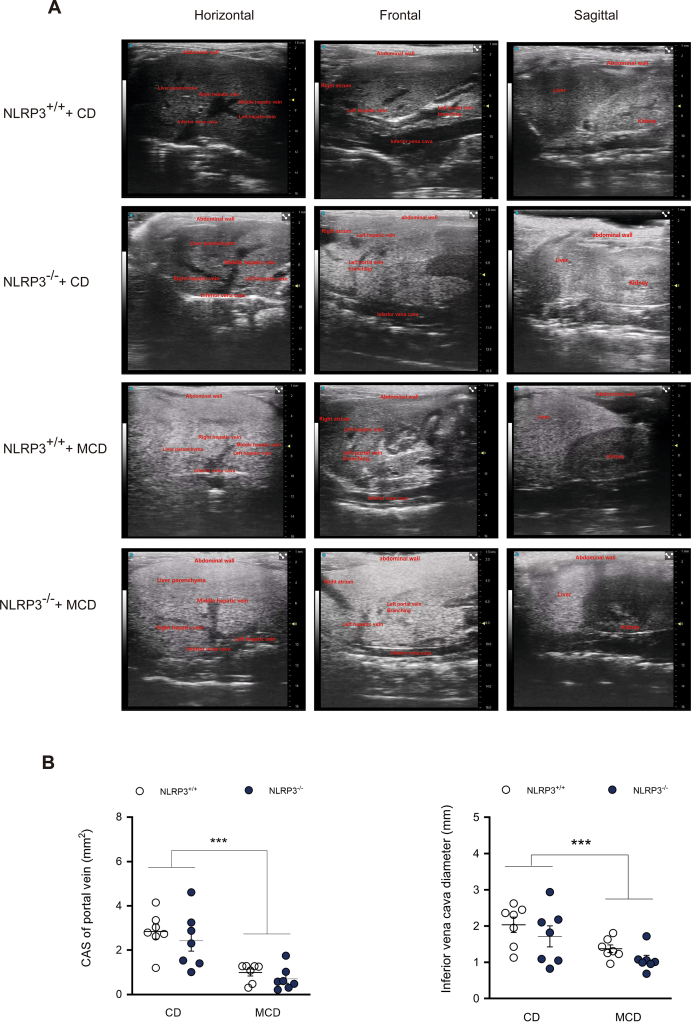


**figs3:**
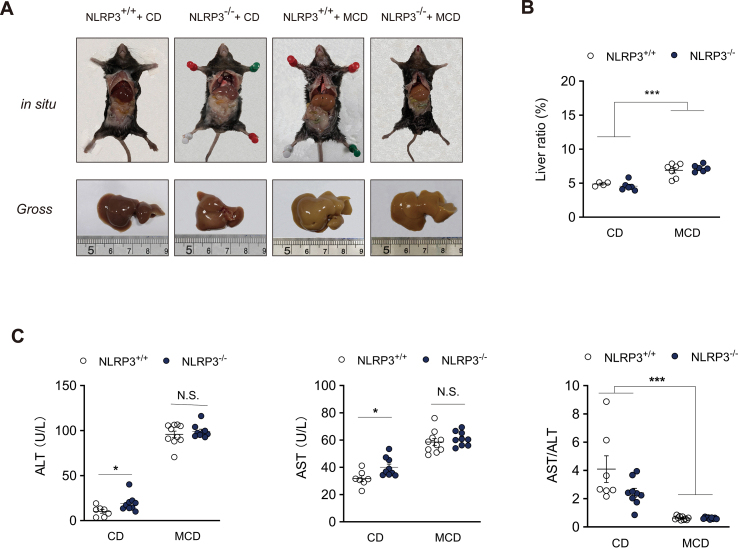


**figs4:**