# Supplementary material for: NLRP3 deficiency reprograms hepatic glucose metabolism but fails to ameliorate hepatic inflammation and fibrosis in the MCD model
Source: Genes Dis. 2025 Nov 6;13(5):101921. doi: 10.1016/j.gendis.2025.101921 (PMC13122682; doi:10.1016/j.gendis.2025.101921)
Supplement: Multimedia component 1 [file mmc1.docx]

**Materials and Methods**

**1 Animal**

Eight-week-old wild-type (C57BL/6J, WT) and NLRP3 knockout (C57BL/6J, Nlrp3^-/-^) male mice were procured from Nanjing GemPharmatech company. Nlrp3 (gene ID: 216799) whole-body knockout mice were generated by deleting exon 3~4 of Nlrp3 using the CRISPR–Cas9 system (GemPharmatech). The sgRNA sequence was as follows: sgRNA1 (matches forward strand of gene): GCTTAGGCATAGTTATCCATGGG; sgRNA2 (matches reverse strand of gene): ACACCATCGTCTCAGAACGACGG. For genotyping, genomic DNA was extracted from mouse tail biopsy samples, and subjected to standard genotyping PCR, Forward, CCACTTGCATTGTATGTTGCCTC, Reverse, TGTAGCGACTGTTGAGGTCCACA. PCR amplification products were as follows: WT allele = 0bp, delete allele = 466 bp. All experimental protocols were approved by the ethic committee of East China Normal University, following the Guidelines of Animal Experiment set by the Bureau of Sciences and Techniques of Shanghai, China [NO. m20210259].

**2 Preparation of the MCD-induced liver fibrosis model**

Mice were maintained on a 12 h light/dark cycle and had unrestricted access to rodent chow diet. At 8 weeks of age, WT and NLRP3^-/-^ mice were housed 4-5 per cage and fed either a 60 kcal % fat diet high in saturated fat (HFMCD; D10080402, Xietong Bio-engineering, China) for 6 weeks. Diet compositions are detailed in Table S1.

**3 Body weight and body composition**

Body weights were recorded weekly. Fat and fat-free mass measurements were obtained by Magnetic Resonance Imaging (MRI) ((AccuFat-1050, MAG-MED) 3 days prior to euthanasia.

**4 Liver B-mode ultrasound imaging**

The mice were evaluated using ultrasound (Vevo 2100, 30-MHz; VisualSonics). Animals were placed in a chamber containing inhaled isoflurane and monitored until full anesthesia was achieved (R640, RWD). The fur on the abdomen was removed using a depilatory cream. During the entire imaging process, the mice were positioned on a warmed platform to track the body temperature, heart rate and respiration. Hepatic/renal and maximum liver planes were analyzed. Average intensity/mm^2 (^a.u.) was calculated by taking the average intensity of three different areas of each plane. All ultrasound images were captured using the same settings (frequency = 30 MHz, gain = 28 dB, depth = 12 mm, dynamic range = 60 dB, width = 15.36 mm, persistence = off, sensitivity = high) from the same machine and completed by the same technician, who was blinded to this study ^1^.

**5 Glucose, insulin and pyruvate tolerance test**

For Pyruvate tolerance test (PTT), fasted for 16 h, and injected intraperitoneally with pyruvate (0.75 mg/kg body weight in saline). Blood glucose levels were measured from the tail vein at indicated times using a Freestyle glucometer. For Glucose tolerance test (GTT) and Insulin tolerance tests (ITT) were done with an intraperitoneal injection of insulin (3 units/kg body weight, i.p.; HI0219, Lilly Egypt) into mice after 6 h of fasting. Fasting blood glucose levels were measured after an overnight fast of 8 hours. Blood glucose concentrations were measured with an Accu-Check glucometer (Sinocare, China).

**6 Liver triglyceride (TG) and hepatocyte lactate assays**

To measure liver triglyceride contents, liver tissues or cell were homogenized in 5% NP40 in PBS. Tissue or cell lysates were centrifuged and the supernatant liquid were obtained and triglyceride or lactate levels were determined by Triglycerides kit (BioVision, K622) or Lactate kit (BioVision, K627) and normalized to liver protein content using BCA protein assay kit (Beyotime, P0012).

**7 Serum biochemistry and ELISA**

Serum IV-C, M2BPGi, HA were measured with ELISA kits (Mlbio, China), Liver tissue was homogenized in PBS to extract the extracellular matrix (ECM), and the levels of IL-1β (Mlbio, China) and IL-18 (Mlbio, China) were normalized to total protein concentrations in the homogenate. Experimental procedures were performed according to the manufacturer’s instructions Serum alanine aminotransferase or aspartate aminotransferase concentrations were measured with a Fuji Dri-Chem 3500 biochemistry analyzer (FujiFilm).

**8 Metabolomics analysis**

Metabolomics analysis was performed in house or with the assistance from Shanghai Biotree Biotech. Metabolites in liver tissues were measured by capillary electrophoresis time of flight mass spectrometry (CE–TOFMS) using the Agilent 7100 CE Capillary Electrophoresis system equipped with an Agilent 6230 Time-of-Flight mass spectrometer (Agilent Technologies) according to previously reported methods. In brief, snap-frozen liver tissue samples were homogenized with 500 μl of methanol containing internal standards (H3304-1002, Human Metabolome Technology) and 500 μl of chloroform and 200 μl of ultrapure water (50% v/v) were subsequently added into the sample solution. The solution was centrifuged at 2,300g for 5 min at 4 °C to remove debris. Subsequently, 300 μl of the aqueous fraction was centrifugally filtered through a 5 kDa cut-off filter using an Ultrafree MC-PLHCC filter (UFC3LCCNB_HMT, Human Metabolome Technology). The filtrate was dried by a dryer for 180 min and dissolved in 25 μl of both Milli-Q water-containing reference compounds (H3304-1004, Human Metabolome Technology). The amount of all anion- and cation-charged metabolites in the samples was measured by CE−TOFMS. For analysis of cationic metabolites, a fused silica capillary (H3305-1002, 50 μm internal diameter × 80 cm total length, Human Metabolome Technologies) with a cation buffer solution (H3301-1001, Human Metabolome Technologies) as the electrolyte was used. The sample was injected at a pressure of 50 mbar for 10s. To determine anionic metabolites, a chemically coated cationic polymer capillary (50 µm internal diameter × 80 cm total length, H3305-1002, Human Metabolome Technologies) was used with an anion buffer solution (H3302-1021, Human Metabolome Technology) as the electrolyte. The sample was injected at a pressure of 50 mbar for 25 s. For measuring cation- and anion-charged metabolites, the CE capillary was preconditioned by flushing the running electrolyte for 3 or 4 min before sample injection. Each sample was inserted with a pressure injection of 50 mbar for 10 s under 20 °C of the capillary temperature and 27 kV and 30 kV applied voltage for measurement of cation- and anion-charged metabolites, respectively. Sheath liquid (H3301-1020, Human Metabolome Technology) was delivered at 10 µl min−1 to the CE interface by a pump using the Agilent 1200 series pump equipped with a sheath flow splitter. Electrospray ionization–TOFMS was conducted in positive-ion mode with 4 kV capillary voltages for cationic metabolites and was set in the negative-ion mode with 3.5 kV for anionic metabolites.

The spectrometer was scanned from m/z 50 to 1,000 at 1.5 cycles per second during separation and detection. An automatic recalibration of each acquired spectrum was performed using the masses of reference standards. For the CE–TOFMS system control and data acquisition, we used an Agilent MassHunter software for TOFMS (Agilent Technologies). All target metabolites were identified by matching their m/z values and migration times with the normalized m/z values and migration times of corresponding authentic standard compounds. Data were quantified by comparing the ratio of analyte peak area/internal standard peak area of sample solution to that of the reference standard mixture. The quantitative formula was as follows: X nmol mg−1 = (sample relative area)/(standard relative area) × (concentration of internal standard in methanol: 50 µM) × (amount of methanol for metabolite isolation (500 µl)) × 1/(sample weight) × (concentration of metabolite in the reference standard mixture (50 µM))/(concentration of internal standard in the reference standard mixture (100 µM)). The sample relative area = (metabolite peak area in the sample solution)/(internal standard area in the sample solution). The standard relative area = (metabolite peak area in the reference standard mixture)/(internal standard area in the reference standard mixture). Chromatograms of each metabolite were extracted and quantified using the Agilent MassHunter Qualitative Analysis software (Agilent Technologies). For lipidomics analysis, 50 μl mouse serum sample was diluted into extraction buffer (isopropyl alcohol (CAEQ-4-013493-4000, CNW Technologies): n-hexane (CAEQ-4-011518-4000, CNW Technologies) = 2:3 (v:v) with internal standards (CDAA252795, ANPEL). Samples were sonicated in an ice-water bath for 5 min, then centrifuged at 4 °C for 15 min at 12,000 rpm. Then, 400 μl of supernatant was dried in a speed vac, stored under nitrogen and reconstituted in 160 μl of n-hexane. After centrifugation at 12,000 rpm for 5 min, the supernatant was projected for gas chromatography–mass spectrometry (5977B, Agilent) for further analysis.

**9 PCR array and qPCR**

Bilateral glucose metabolism was carefully dissected according to standard stereotaxic coordinates. Total RNA was isolated with the nRNAeasy™ Animal RNA Isolation Kit, reverse-transcribed into cDNA (BeyoRT™ II M MLV Kit), and analyzed Via real- time PCR (QuantStudio 3/5 System) using Wcgene Biotech’s mRNA qPCR mix. A mouse glucose metabolism microarray assessed expression profiles of 87genes (Table S2). The thermocycling conditions were as follows: initial denaturation at 95 °C for 30 s, followed by 40 cycles of 95 °C for 5 s and 60 °C for 30 s. Melting curve analysis was conducted to confirm amplification specificity. All reactions were performed in technical triplicates. The Actb gene was used as an internal control for normalization. Relative gene expression was calculated using the 2^(-ΔΔCt) method. PCR Arry data were processed using Wcgene Biotech’s software. The sequence for primers used was as follows: *Ldha,* forward, TGTCTCCAGCAAAGACTACTGT, reverse, GACTGTACTTGACAATGTTGGGA, *Ldha* forward, CATTGCGTCCGTTGCAGATG, Reverse, GGAGGAACAAGCTCCCGTG.

**10 Histology, staining and Immunogen Fluoresce**

Dissected liver tissues were fixed in 10% neutral buffered formalin (Sigma-Aldrich, HT501128-4L) and embedded in paraffin. 5μm tissue sections were stained with Hematoxylin and eosin (Beyotime, C0105S) according to manufacturer's instructions. The images were acquired by optical microscope (Nikon) using a 20x objective. NES sizes were quantified by ImageJ. Evaluation of NAFLD activity score (NAS) has been described before. We receive information for steatosis (<5% = 0, 5–33% = 1, 33–66% = 2, >66% = 3), lobular inflammation (none = 0, <2 foci = 1, 2–4 foci = 2, >4 foci = 3), and locular ballooning (none = 0, few = 1, prominent = 2). The NAS is the unweighted sum of steatosis, lobular inflammation, and hepatocellular ballooning scores ^2^.

For PAS staining, sections were incubated with 1% periodic acid solution for 10 minutes at room temperature, followed by two washes in distilled water. The sections were then stained with Schiff’s reagent for 15 minutes in the dark and rinsed thoroughly in running tap water for 5–10 minutes until the background cleared. Nuclei were counterstained with hematoxylin for 1 minute, then the sections were dehydrated, cleared in xylene, and mounted with coverslips.

For PSR staining, slides were stained in 0.1% Sirius Red solution dissolved in saturated picric acid for 60 minutes at room temperature. Excess stain was removed by rinsing briefly in 0.5% acetic acid twice. The slides were then dehydrated through graded ethanol, cleared in xylene, and mounted. Collagen fibers appeared red under bright-field microscopy.

Paraffin-embedded liver tissue sections (4–6 μm) were deparaffinized, rehydrated, and subjected to antigen retrieval in citrate buffer (pH 6.0) at 95 °C for 15 minutes. Endogenous peroxidase activity was blocked with 3% hydrogen peroxide for 10 minutes, followed by blocking with 5% normal goat serum for 30 minutes at room temperature.

For Immunohistochemistry, sections were incubated with F4/80 (70076, CST) overnight at 4 °C. After washing, HRP-conjugated secondary antibodies were applied for 30 minutes at room temperature. Signal was developed using DAB substrate, and nuclei were counterstained with hematoxylin. Slides were dehydrated, mounted, and imaged using a bright-field microscope.

For immunogen fluoresce, the paraffin-embedded liver tissue sections were cut into 10 μm coronal sections and used for immunofluorescence staining. These prepared sections were blocked with goat serum and incubated with α-SMA (ab5694, Abcam) overnight at 4 °C, followed by fluorescence conjugated secondary antibody (Servicebio, Wuhan, China). The sections then added DAPI (D9542, Sigma) and were incubated in dark for 10min, followed by antigen retrieval with EDTA antigen retrieval buffer (1 mM EDTA, pH 8.0). The immunofluorescence was captured by using a Nikon Eclipse C1 microscope.

**11 Isolation and culture of mouse primary hepatocytes**

Primary mouse hepatocytes were cultured as previously described (4). In brief, 5- to 6-week-old male C57BL/6 mice were anesthetized with 10% chloral hydrate, and a catheter was placed in the inferior vena cava. The liver was perfused with 1 mL heparin (320 μ/mL), 40 mL solution I (Krebs’s solution and 0.1 mmol/L EGTA), and 30 mL solution II (Krebs’s solution, 2.74 mmol/L CaCl2, and 0.05% collagenase I), respectively. The perfused liver was passed through a 400-μm screening size filter by flushing with RPMI 1640 medium. The hepatocytes were collected by centrifuge at 50g for 2 min. Hepatocytes were resuspended with RPMI 1640 medium and plated in six-well plates for experiments after three washes with RPMI 1640.

**12 Cell glycolysis capacity test**

Assays were performed using the Seahorse XFe24 analyzer (Seahorse Bioscience, Agilent) according to the manufacturer’s instructions. Briefly, 6 x10^4^ cells/well were seeded in a 24-well XF cell culture microplate in growth medium 24 h before assay. ECAR was measured with an XF24 analyzer in XF base medium (pH 7.4) containing 1 mM glutamine following sequential additions of glucose (10 mM), oligomycin (1 μM) and 2-DG (50 mM). Data were analyzed by the Seahorse XF Glycolysis Stress Test Report Generator package.

**13 Cell mitochondria stress test**

Mitochondrial respiratory function was analyzed using the Seahorse XFe24 Analyzer (Agilent Technologies). Primary hepatocytes were isolated from mouse livers and seeded at a density of 6 x10^4^ cells per well in Seahorse XF24 cell culture microplates. After 24 hours of culture, cells were washed and incubated in Seahorse XF DMEM assay medium (pH 7.4) supplemented with 10 mM glucose, 1 mM pyruvate, and 2 mM glutamate for 1 hour at 37°C in a non-CO₂ incubator. The mitochondrial stress test was performed through sequential injection of the following modulators: oligomycin (1.5 μM) to inhibit ATP synthase, FCCP (1.0 μM) to uncouple mitochondrial respiration, and a mixture of rotenone and antimycin A (0.5 μM each) to inhibit electron transport chain complexes I and III. The oxygen consumption rate (OCR) were measured in real-time. Key parameters of mitochondrial function, including basal respiration, ATP production, proton leak, maximal respiration, and spare respiratory capacity, were calculated according to standard protocols.

**14 Metabolic cages**

For metabolic phenotyping, mice were placed in individual metabolic cages in a CLAMS (Comprehensive Laboratory Animal Monitoring System, Columbus Instruments, OH, USA) for two consecutive days, with free access to food and water. Food and water consumption, oxygen consumption, carbon dioxide production, and movement were monitored in real-time. Data analysis was performed using their application from the company.

**15 ATP/ADP Measurement in Primary Hepatocytes**

ATP and ADP levels were quantified in primary hepatocytes using a luminescence-based assay kit (Elabscience, E-BC-F004). Cells were lysed with boiling buffer to inactivate nucleotidases. Luminescence was measured directly for ATP. For ADP, lysates were incubated with ADP-to-ATP conversion enzyme before measurement. Values were normalized to protein concentration.

**16 NAD+ and NADH level measurement**

The NAD⁺/NADH ratio was determined using a WST-8-based commercial assay kit (Beyotime #S0175). Briefly, primary hepatocytes or liver tissues were homogenized in extraction buffer. To separately quantify NAD⁺ and NADH, samples were split and processed with specific treatments: one aliquot was heated at 60°C for 30 min to decompose NAD⁺ (for NADH measurement), while the other remained untreated (for total NAD(H) measurement). The WST-8 reagent was added to samples and incubated at 37°C for 30 min. Absorbance was measured at 450 nm using a microplate reader. The NAD⁺ content was calculated by subtracting NADH from total NAD(H). Values were normalized to protein concentration.

**17 Western Blot**

Proteins were extracted from liver tissues using RIPA lysis buffer containing protease and phosphatase inhibitors. Protein concentrations were determined by BCA assay. Equal amounts of protein (20-30 μg) were separated by SDS-PAGE and transferred to PVDF membranes. After blocking with 5% non-fat milk, membranes were incubated with primary antibodies overnight at 4°C, followed by HRP-conjugated secondary antibodies. Protein bands were visualized using ECL substrate and quantified by ImageJ software. β-Actin or GAPDH was used as a loading control. Antibody information: NLRP3 antibody(CST, #15101), β-actin(CST, #4970).

**18 Stataics analysis**

Statistical analysis was performed using GraphPad Prism (version 10.4, La Jolla, CA, USA). A two-way analysis of variance (ANOVA) with Turkey methods was used for the evaluation of differences among the four groups. PTT data were analyzed using repeated-measures ANOVA. Post hoc pairwise comparisons for significant main effects were conducted using Tukey's honestly significant difference (HSD) test to control for multiple comparisons. Genes with a fold change greater than 2 or less than 0.5 were selected for KEGG analysis.

**References**

1. Wear KA, Han A, Rubin JM, et al. US Backscatter for Liver Fat Quantification: An AIUM-RSNA QIBA Pulse-Echo Quantitative Ultrasound Initiative. Radiology. 2022;305(3):526-537.
2. Kleiner DE, Brunt EM, Van Natta M, et al. Design and validation of a histological scoring system for nonalcoholic fatty liver disease. Hepatology. 2005;41(6):1313-1321. doi:10.1002/hep.20701

| **Table S1. HFMCD Diet compositions** | | |
| --- | --- | --- |
| **Ingredient** | **gm** | **kcal** |
| L-Methionine | 0 | 0 |
| L-Alanine | 5.1 | 20.4 |
| L-Arginine | 6 | 24 |
| L-Aspartic Acid | 12.1 | 48.4 |
| L-Cystine | 4.2 | 16.8 |
| L-Glutamic Acid | 38.2 | 152.8 |
| Glycine | 3 | 12 |
| L-Histidine | 4.6 | 18.4 |
| L-Isoleucine | 7.6 | 30.4 |
| L-Leucine | 15.8 | 63.2 |
| L-Lysine | 13.2 | 52.8 |
| L-Phenylalanine | 8.4 | 33.6 |
| L-Proline | 17.8 | 71.2 |
| L-Serine | 10 | 40 |
| L-Threonine | 7.2 | 28.8 |
| L-Tryptophan | 2.1 | 8.4 |
| L-Tyrosine | 9.2 | 36.8 |
| L-Valine | 9.3 | 37.2 |
| Total L-Amino Acids | 173.8 | 695.2 |
| Sucrose | 68.8 | 275.2 |
| Corn Starch | 0 | 0 |
| Maltodextrin | 130.1 | 520.4 |
| Cellulose | 50 | 0 |
| Soybean Oil | 25 | 225 |
| Lord | 245 | 2205 |
| Mineral Mix S10026B | 50 | 0 |
| Vitamin Mix V10001C | 1 | 4 |
| Choline Bitartrate | 0 | 0 |
| Sodium Bicarbonate | 7.5 | 0 |
| FD&C Yellow Dye #5 | 0.03 | 0 |
| FD&C Red Dye #40 | 0.03 | 0 |
| **Total** | 755.26 | 3924.8 |

| **Table S2. Glucose metabolism related genes in the PCR array and the assay results** | | | | |
| --- | --- | --- | --- | --- |
| **Classification** | **Gene name** | **Nlrp3^-/-^ / Nlrp3^+/+^** | **Log2(FC)** | **P-value** |
| Glycolysis | Aldoa | 1.206167359 | 0.270430099 | 0.605190717 |
|  | Aldob | 1.28486507 | 0.361616863 | 0.183585051 |
|  | Aldoc | 1.279715029 | 0.355822582 | 0.516868367 |
|  | Bpgm | 0.935242715 | -0.096587271 | 0.560215198 |
|  | Eno1 | 1.122914241 | 0.16724775 | 0.264489655 |
|  | Eno2 | 0.488094954 | -1.034766258 | 0.341895198 |
|  | Eno3 | 1.331399182 | 0.412943187 | 0.38961452 |
|  | Galm | 1.213479346 | 0.279149553 | 0.133519283 |
|  | Gapdhs | 1.625411799 | 0.700805272 | 0.102075321 |
|  | Gck | 1.283020305 | 0.359544003 | 0.007407675 |
|  | Gpi1 | 1.409958517 | 0.495652717 | 0.18135648 |
|  | Hk2 | #DIV/0! | #DIV/0! | #DIV/0! |
|  | Hk3 | 2.418864438 | 1.274329918 | 0.026165365 |
|  | Ldha | 1.28305959 | 0.359588176 | 0.01494718 |
|  | Pfkl | 3.712551195 | 1.89241092 | 0.010121551 |
|  | Pgam2 | 0.963101799 | -0.054239797 | 0.812349558 |
|  | Pgk1 | 1.059887061 | 0.083910543 | 0.561804243 |
|  | Pgk2 | 1.028345431 | 0.040324961 | 0.768538262 |
|  | Pklr | 1.050826654 | 0.0715247 | 0.434656593 |
|  | Tpi1 | 1.545798308 | 0.628352092 | 0.132686799 |
| Glycogen regulation | Gsk3a | 1.39406318 | 0.479295947 | 0.236046434 |
|  | Gsk3b | 1.530979765 | 0.614455214 | 0.097410607 |
|  | Phka1 | 1.007469339 | 0.010735933 | 0.951744869 |
|  | Phkb | 3.609719465 | 1.85188672 | 0.422555075 |
|  | Phkg1 | 0.024428766 | -5.355275225 | 0.289180821 |
|  | Phkg2 | 6.894878361 | 2.7855251 | 0.483946169 |
| Glycogen synthesis | Gbe1 | 9.104969399 | 3.186654168 | 0.255460811 |
|  | Gys1 | #DIV/0! | #DIV/0! | #DIV/0! |
|  | Gys2 | 1.165762275 | 0.22127362 | 0.08525497 |
|  | Ugp2 | 0.989848565 | -0.014720269 | 0.935914366 |
| gluconeogenesis | Fbp1 | 1.288571667 | 0.365772778 | 0.062547015 |
|  | G6pc | 3.334582192 | 1.737506009 | 0.011231863 |
|  | G6pc3 | 1.376644355 | 0.461155899 | 0.042032698 |
| Pentose Phosphate Pathway | Agl | 1.108021389 | 0.147985732 | 0.108326008 |
|  | Pgm1 | 1.064155729 | 0.089709291 | 0.447149621 |
|  | Pgm2 | 0.990498652 | -0.013773082 | 0.878272267 |
|  | Pgm3 | #DIV/0! | #DIV/0! | #DIV/0! |
|  | Pygl | 1.435435189 | 0.521488194 | 0.037738507 |
|  | Pygm | 1.087571211 | 0.121109867 | 0.200634139 |
|  | G6pdx | #DIV/0! | #DIV/0! | #DIV/0! |
|  | H6pd | 1.122384663 | 0.166567201 | 0.117525026 |
|  | Prps1 | 1.050895409 | 0.071619092 | 0.441139445 |
|  | Prps1l1 | 1.004249226 | 0.006117349 | 0.962462355 |
|  | Prps2 | 1.198361332 | 0.261062978 | 0.592904578 |
|  | Rbks | 1.03536394 | 0.050137978 | 0.55001345 |
|  | Rpe | #DIV/0! | #DIV/0! | #DIV/0! |
|  | Rpia | #DIV/0! | #DIV/0! | #DIV/0! |
|  | Taldo1 | 1.145996304 | 0.196602391 | 0.046960837 |
|  | Tkt | 3.051605647 | 1.609568537 | 0.16487321 |
| Tricarboxylic Acid Cycle | Acly | 1.222998641 | 0.290422801 | 0.610565093 |
|  | Aco1 | 1.100565989 | 0.13824565 | 0.270894316 |
|  | Aco2 | 0.799603188 | -0.322643871 | 0.647986848 |
|  | Cs | 1.079173027 | 0.109926194 | 0.423180879 |
|  | Dlat | 6.494735491 | 2.699270771 | 0.341046803 |
|  | Dld | 2.383275222 | 1.252945564 | 0.182439055 |
|  | Dlst | 2.517552287 | 1.332021742 | 0.273149099 |
|  | Fh1 | 1.32884654 | 0.410174507 | 0.093322994 |
|  | Idh3a | 0.271017695 | -1.883541047 | 0.431602238 |
|  | Idh3b | 1.193811831 | 0.255575457 | 0.446298975 |
|  | Idh3g | 0.807652638 | -0.308193155 | 0.631172934 |
|  | Mdh1 | #DIV/0! | #DIV/0! | #DIV/0! |
|  | Mdh1b | 1.209676289 | 0.274621032 | 0.518345589 |
|  | Mdh2 | 1.100225516 | 0.137799267 | 0.22363992 |
|  | Ogdh | 1.109340743 | 0.14970257 | 0.282492611 |
|  | Pck1 | 1.011231215 | 0.016112903 | 0.905115797 |
|  | Pck2 | #DIV/0! | #DIV/0! | #DIV/0! |
|  | Pcx | 2.612638559 | 1.385507551 | 0.054806402 |
|  | Pdha1 | 1.289024614 | 0.366279813 | 0.037609534 |
|  | Pdhb | 1.134018499 | 0.181444175 | 0.134977986 |
|  | Sdha | #DIV/0! | #DIV/0! | #DIV/0! |
|  | Sdhb | 0.699452547 | -0.515701911 | 0.424151549 |
|  | Sdhc | 5.283172544 | 2.401404528 | 0.337521702 |
|  | Sdhd | 0.59751894 | -0.742943651 | 0.592307132 |
|  | Sucla2 | #DIV/0! | #DIV/0! | #DIV/0! |
|  | Suclg1 | 1.080450314 | 0.111632729 | 0.250506264 |
|  | Suclg2 | 1.116863928 | 0.159453427 | 0.199192755 |

**Figure S1**. **Role of NLRP3 deficiency in metabolic physiology in mice and primary hepatocyte**. (A) Generation of Nlrp3⁻/⁻ mice and validation of hepatic knockout efficiency (n = 3 per group). (B) body weight, (C) lean mass and (D) fat mass in Nlrp3⁻/⁻ and control mice (n = 8 per group). (E) skeletal muscle weight in Nlrp3⁻/⁻ and control mice (n = 8 per group). (F) adipose tissue weight in Nlrp3⁻/⁻ and control mice (n = 8 per group). (G) Monitoring of oxygen consumption (VO₂) and (H) carbon dioxide production (VCO₂) in mice over a 48-hour period using metabolic cages (n = 5 per group). (I) Spontaneous locomotor activity and (J) food intake in mice over a 24-hour period (n = 5 per group). (K) Glucose tolerance test and its AUC (n = 7 per group). (L) Insulin tolerance test and its AUC (n = 7 per group). (M)Metabolite abundance (nmol/g) from the target metabolomics data in mice liver tissue (n=3-4 per group). (N)Assessment of non-mitochondrial oxygen consumption and proton leak in Nlrp3⁻/⁻ primary hepatocytes (n = 7 per group). The data were expressed as mean ± standard error. ∗P < 0.05, ∗∗P < 0.01, ∗∗∗P < 0.001 compared to control.

**Figure S2**. **Hepatic ultrasonographic morphology and quantitative analysis in NLRP3 mice with chow diet or MCD.** (A) ultrasound images and (B)its representative data showing hepatic morphology, including echogenicity and vascular structure, across groups (n = 7 per group). The data were expressed as mean ± standard error. ∗P < 0.05, ∗∗P < 0.01, ∗∗∗P < 0.001 compared to control. # P < 0.05 interaction in 2-way ANOVA. Post hoc pairwise comparisons for significant main effects were conducted using Tukey's honestly significant difference (HSD) test to control for multiple comparisons.

**Figure S3**. **Assessment of liver morphology, relative liver weight, and serum injury markers in mice.** (A)Gross liver morphology and (B) liver-to-body weight ratio in Nlrp3⁻/⁻ and littermate controls under MCD or chow diet (n = 5–6 per group). (C) Serum liver injury markers (ALT, AST) in Nlrp3⁻/⁻ and controls on chow or MCD diet (n = 7 per group). The data were expressed as mean ± standard error. ∗P < 0.05, ∗∗P < 0.01, ∗∗∗P < 0.001 compared to control. # P < 0.05 interaction in 2-way ANOVA. Post hoc pairwise comparisons for significant main effects were conducted using Tukey's honestly significant difference (HSD) test to control for multiple comparisons.

**Figure S4**. **Evaluation of hepatic inflammation and fibrosis in NLRP3 mice with chow diet or MCD.** (A) H&E staining, Picrosirius Red (PSR) staining and Immunohistochemical staining for F4/80. Scale bars, 100 μm. (B) NAFLD activity scores (NAS) based on steatosis, inflammation, and ballooning degeneration (n = 3 per group, each sample for 3 replicate). (C) Quantification of hepatic fibrosis by Sirius Red staining (n = 3 per group, each sample for 3 replicate). (D) Quantification of liver extracellular matrix (ECM) fibrosis markers by ELISA (n = 7 per group). (E) Immunofluorescence analysis of hepatic stellate cell (HSC) activation markers in mouse liver sections. (F) Quantification of liver extracellular matrix (ECM) pro-inflammatory cytokines markers by ELISA (n = 7 per group). The data were expressed as mean ± standard error. ∗P < 0.05, ∗∗P < 0.01, ∗∗∗P < 0.001 compared to control. # P < 0.05 interaction in 2-way ANOVA. Post hoc pairwise comparisons for significant main effects were conducted using Tukey's honestly significant difference (HSD) test to control for multiple comparisons.
